# The Experimental and Modeling Study on the Effect of Ethane in Helium-Rich Natural Gas on the Thermodynamic Equilibrium of Hydrate Formation in the Presence of Tetrahydrofuran

**DOI:** 10.3390/molecules30102109

**Published:** 2025-05-09

**Authors:** Zengqi Liu, Rui Wang, Haixin Chen, Zhen Xu, Shiguang Fan, Qiang Sun, Yiwei Wang, Xuqiang Guo

**Affiliations:** 1 State Key Laboratory of Heavy Oil Processing, China University of Petroleum-Beijing at Karamay, Karamay 834000, China; liuzq@cupk.edu.cn (Z.L.); guoxq@cup.edu.cn (X.G.); 2 State Key Laboratory of Heavy Oil Processing, China University of Petroleum (Beijing), Beijing 102249, China

**Keywords:** extracting helium, gas hydrate, ethane, thermodynamic equilibrium

## Abstract

Hydrate-based gas separation (HBGS) is a new method for extracting helium from helium-rich natural gas (HNG). The ethane in HNG affects the thermodynamic equilibrium hydrate formation pressure (*Peq*), and *Peq* is crucial to the application of HBGS for extracting helium. In this work, the *Peq* of the HNGs with different ethane contents (0.5 mol%, 1.0 mol%, and 10 mol%) and the solutions with different tetrahydrofuran (THF) contents (5 wt%, 10 wt%, and 19 mol%) at different temperatures were experimentally investigated through the isothermal pressure search method. Ethane and THF have a competitive effect on hydrate formation. A new thermodynamic model was proposed to predict the *Peq* of different HNG–THF solution systems. The effect of ethane on *Peq* can be quantitatively described, and the *Peq* of HNGs can be accurately predicted by the model in this work. The average relative deviation of the model for predicting *Peq* of HNGs in different THF aqueous solution systems is less than 3%. The results of this study can guide the operating conditions for the optimization of extracting helium from HNGs by the HBGS process.

## 1. Introduction

Helium is the lightest of the noble gases, and the boiling and freezing points of helium are lower than those of any other known substance [1]. Helium is a crucial strategic resource with a wide range of applications in scientific research, aerospace, military, medical, and high-end manufacturing [2]. The total global helium resources are estimated at 5.19 × 10^10^ m^3^, with 88% of these reserves in the United States, Qatar, Algeria, and Russia [3]. Most of these reserves cannot become helium production because extracting helium from helium-rich natural gas (HNG) is the only industrial-scale helium production method [4]. Generally, helium production is maintained at 1.6 × 10^8^ m^3^ (standard state) per year, but helium demand is growing rapidly each year, which only in China is reaching 2.2 × 10^7^ m^3^ (standard state) in 2021 [5]. Therefore, driven by the growing demands for helium production and highly efficient recovery from HNG, the development of innovative helium extraction processes has become a research priority.

HNG is a natural gas with a helium content of 0.3–2%, used as the main feed for helium extraction [6]. The cryogenic separation process is the most used method for extracting helium [7]. The cryogenic helium separation processes are already applied industrially [8,9]. The process simulation suggests that cryogenic helium separation is the most cost-effective helium recovery configuration [10]. The expensive and energy-intensive cryogenic processes limit the cost of helium production [11]. The pressure swing adsorption [12], adsorption [13], and membrane separation [14] are widely anticipated extracting helium processes. The process simulation [15] suggests that membrane separation technologies for helium recovery have the potential to be economically viable. Due to the separation efficiency and costs in industrial applications for extracting helium [16], a novel separation process is expected.

The hydrate-based gas separation (HBGS) process has good potential for helium extraction. The performance of HBGS is not negatively affected by acid gases (like CO_2_ and H_2_S), which can be trapped in gas hydrates [17]. The operating temperature of HBGS approximates room temperature, and its energy consumption is low [18]. It also has a good performance in the separation of natural gas [19]. Compared to cryogenic separation, the approximate room temperature has no risk of allowing carbon dioxide to solidify [20]. The conditions for the formation of nitrogen hydrates are harsher (higher pressure and lower temperature than methane), and the energy consumption of HNG containing more nitrogen will be greatly increased by HBGS [21].

**Figure 1:**
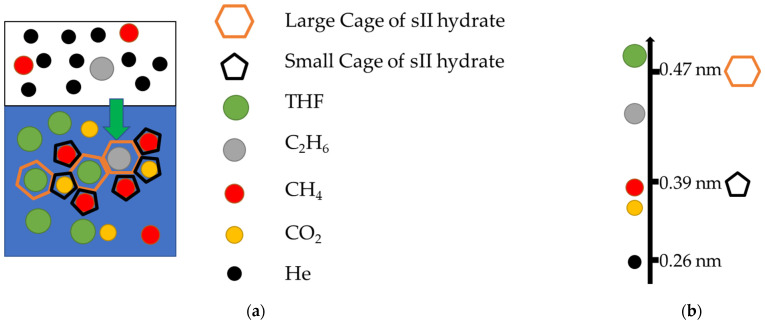
The schematic of the differences in gas molecules for hydrate formation. The blue background is the liquid phase, and the white background is the gas phase [23]. (**a**) The schematic of HBGS. (**b**) Hydrate cage (sII) and gas molecule sizes.

Gas hydrates are non-stoichiometric cage-like crystals formed by water and small gas molecules [22]. Generally, there are three clathrate structures, which are structure I (sI), structure II (sII), and structure H (sH) [23]. The sII hydrate (24 Gas·136 H_2_O) with 8 larger cages (5^12^6^4^, a hexahedron with 12 pentagons and 4 hexagons) and 16 small cages (5^12^, a dodecahedron with 12 pentagons). The difference among the diameters of different gas molecules leads to the difference among the thermodynamic conditions required by different gas hydrates, which are shown in Figure 1 [23]. When the diameters of gas molecules are similar to cages, the gas molecules can be trapped in the cages, like CH_4_ [24], CO_2_ [25], C_2_H_6_ [26], and tetrahydrofuran (THF) [27]. When the diameter is smaller than the cages, the gas molecules can hardly be trapped in the cages, like hydrogen [28,29] and helium [30]. Consequently, the HBGS process for helium extraction holds significant research value.

In cryogenic helium separation processes, a deep knowledge of the thermodynamics of the treated mixture is needed [20]. In HBGS, a deep knowledge of the thermodynamic equilibrium hydrate formation pressure (*Peq*) of gas mixture hydrate is also needed. The hydrate formation condition is crucial to the HBGS that extracts helium from HNGs [29]. The *Peq* is the lowest pressure for hydrate formation at a certain temperature. The lower *Peq* led to lower energy consumption [31]. The thermodynamic promoters, like THF, can efficiently decrease the number *Peq* of HNGs [32]. Previous research [30] proved that THF can significantly decrease the hydrate formation of *Peq* binary gas (CH_4_ and helium) and ternary gas (CH_4_, helium, and CO_2_). For that reason, THF was chosen as a thermodynamic promoter in this work.

Ethane is a common component in natural gas [4]. Both ethane and THF can form sII hydrate with methane [33]. When ethane and THF are present in the same system, both THF and ethane can be trapped in the large cages of sII hydrate. Sun et al. [34] found that competition between THF and ethane exists in the methane-ethane-THF system in the presence of high THF concentrations. Lee et al. [35] found that the effect of ethane is different between high THF and low THF concentrations in the methane–ethane–propane–THF system. Therefore, further elucidating the effects of ethane and THF on *Peq* is critical for extracting helium from HNGs.

To reveal the effects of ethane and THF on the Peq of HNG, the *Peq* of HNG containing ethane and THF was experimentally investigated and accurately predicted by a new thermodynamic model in this work. This model quantitatively describes the effects of ethane on *Peq*. This is the first thermodynamic model that accounts for the effects of ethane in HNGs on *Peq*. The investigation and model validate the technical feasibility of helium extraction by HBGS. It provides new insight into sII hydrate formation and guides the operating conditions for optimization of extracting helium from HNGs by the HBGS process.

## 2. Results

To show the effects of THF on hydrate formation, the initial concentrations of THF (*w*) in aqueous solutions are 5 wt%, 10 wt%, and 19 wt%. To show the effects of ethane on hydrate formation, the *Peq* of HNGs (mixture of methane, ethane, carbon dioxide, and helium) systems were measured in this work, and the compositions of the experimental gases are listed in Table 1.

As listed in Table 1, the compositions of the mixture gases refer to the actual HNG from Aksu, Xinjiang, China (Aksu gas) [30]. And the different contents of ethane are introduced in this work. Since the methane content in natural gas is above 90 mol% [4], the ethane contents in gas mixtures of methane and ethane are set at 1 mol% and 10 mol% to show the effects of ethane on hydrate formation. The ethane contents (mole ratio) in gases 3~5 (*y*_2_) are 0.5, 1, and 5 mol% to show the effects of ethane on hydrate formation. The contents of helium (*y*_3_) and carbon dioxide (*y*_4_) are 4 and 0.4 mol%, respectively, which is the same as the Aksu gas.

### 2.1. Peq in the THF–Methane–Ethane System

**Figure 2:**
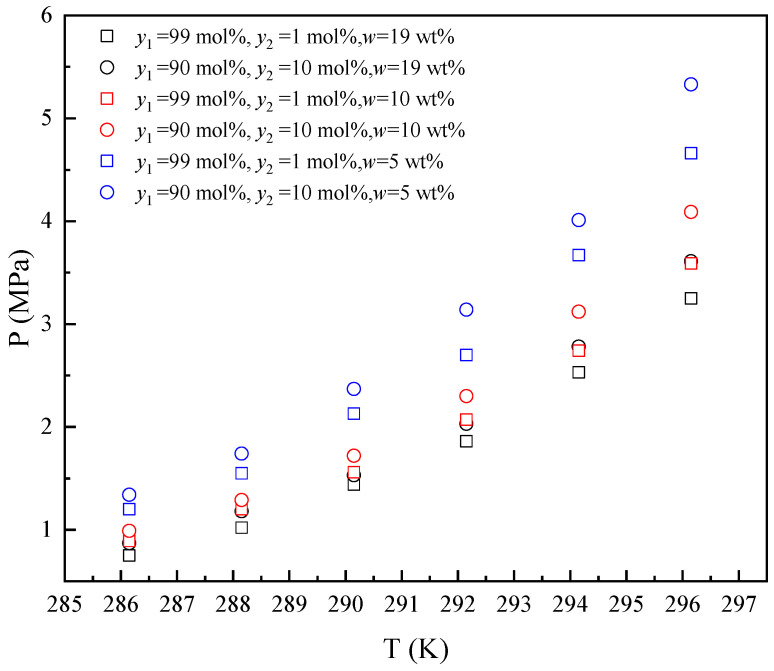
Equilibrium hydrate formation conditions for THF–methane (1)–ethane (2) system.

Since the methane content in natural gas is above 90 mol% [4], the ethane contents in gas mixtures of methane and ethane are set at 1 mol% and 10 mol% to show the effects of ethane on hydrate formation. The experimental investigations on *Peq* were conducted across a controlled temperature range (286.15–296.15 K) for the THF–methane–ethane system in the presence of 5 wt%, 10 wt%, and 19 wt% THF. The results are shown in Figure 2.

As illustrated in Figure 2, the *Peq* increased with the increase in temperature from 286.15 K to 296.15 K in the presence of different THF concentrations. The *Peq* decreased with the increase in the mole fraction of ethane in the gas mixture (*y*_2_ = 1 mol% and *y*_2_ = 10 mol%) in each initial concentration of THF in aqueous solutions. The variation tendency of *Peq* with ethane content is the same as in the literature [34]. As the ethane content in the gas phase increases, the methane content decreases and reduces the fugacity of methane, thus increasing the *Peq*. The *Peq* decreases with the increased THF concentration in aqueous solutions from 5 wt% to 19 wt% in each gas mixture. THF has a promoting effect on *Peq*, and the higher THF concentration (from 5 wt% to 19 wt%) has a stronger promotion effect on *Peq*.

### 2.2. Peq in the THF–Methane–Ethane–Helium–Carbon Dioxide System

**Figure 3:**
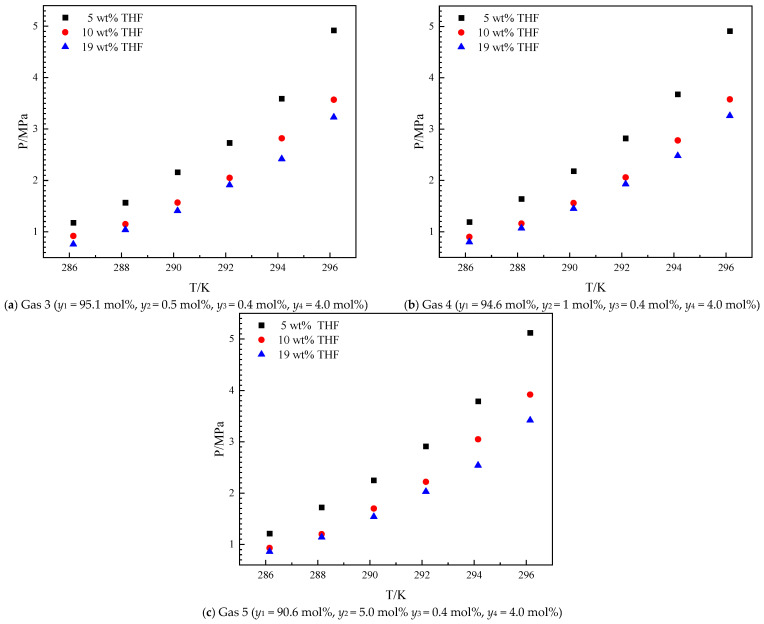
Equilibrium hydrate formation conditions for the THF–methane (1)–ethane (2)–helium (3)–carbon dioxide (4) system.

To further explore the effects of ethane in HNGs on hydrate formation, helium and carbon dioxide are introduced into the system, which refers to the Aksu gas [30]. The ethane contents in HNGs are 0.5, 1, and 5 mol% to show the effects of ethane on hydrate formation. The experimental investigations on *Peq* were conducted across a controlled temperature range (286.15–296.15 K) for the methane–ethane–helium–THF system in the presence of 5 wt%, 10 wt%, and 19 wt% THF. The results are shown in Figure 3.

As illustrated in Figure 3, the *Peq* increased with the increase in temperature from 286.15 K to 296.15 K in the presence of different THF concentrations. The introduction of carbon dioxide and helium does not change the trend of *Peq* with the increase in *T* and initial concentration of THF in aqueous solutions. The variation tendency of *Peq* with ethane content is the same as the THF–methane–ethane system. The Gas 4 and Gas 1 systems have the same ethane content (*y*_2_ = 1 mol%). The *Peq* of the Gas 4 approach is different from that of the Gas 1 with different temperatures and THF concentrations. In the Gas 4 system, helium can hardly be trapped in THF hydrates, but carbon dioxide can be trapped in THF hydrates [36]. 4 mol% carbon dioxide content has a weak effect on *P_eq_* for the THF-methane-helium-carbon dioxide system, which has been discussed in our previous work [30]. The effects of the introduction of ethane will be discussed in Section 3.2. The *Peq* increased with the increased THF concentration in aqueous solutions from 5 wt% to 19 wt% in each gas mixture. THF has a promoting effect on *Peq*, and the higher THF concentration (from 5 wt% to 19 wt%) has a stronger promotion effect on *Peq*. The promoting effect of THF in the THF–methane–ethane–helium–carbon dioxide system is similar to that in the THF–methane–ethane system and other THF-containing systems [37,38].

## 3. Discussion

To quantify the effects on hydrate formation, the differences in *Peq* (Δ*P*%) were proposed as follows [30]:

(1) $$\Delta P\%=\frac{P_{i}-P_{0}}{P_{0}}\times 100\%$$

where $P_{0}$ and $P_{i}$ are experimental *Peq* without and with ethane at the same experimental temperature, respectively. In the same concentration of THF and content of ethane, the average (*A*) and standard deviation (*SD*) of the Δ*P*% were described as follows:

(2) $$A=\frac{\sum_{T}\left({\Delta P\%}_{T}\right)}{n}$$

(3) $$SD=\sqrt{\frac{\sum_{T}{\left({\Delta P\%}_{T}-A\right)}^{2}}{n}}$$

where ${\Delta P\%}_{T}$ is the ΔP% in the experimental temperature (*T*) from 286.15 to 296.15 K. *n* is the total experimental number in the same concentration of THF and content of ethane.

### 3.1. The Effects of Ethane on Hydrate Formation in the THF–Methane–Ethane System

**Figure 4:**
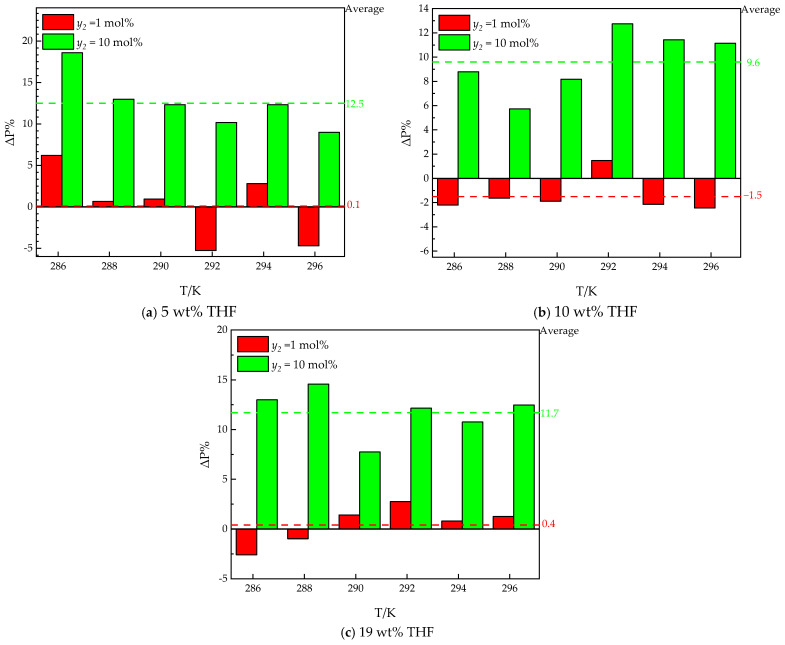
The differences in *Peq* between THF–methane (1) [29] and THF–methane (1)–ethane (2) systems.

To study the effects of ethane on hydrate formation, the *Peq* in THF–methane and THF–methane–helium systems from the literature [30] are introduced for comparison. The results are shown in Figure 4.

**Figure 5:**
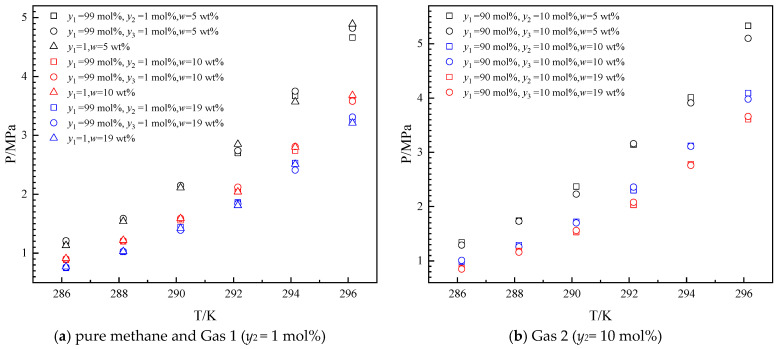
Equilibrium hydrate formation conditions for THF–methane (1) [30], THF–methane (1)–ethane (2), and THF–methane (1)–helium (3) systems [30].

As illustrated in Figure 5, the *Peq* of the THF–methane–ethane system is close to the *Peq* of the THF–methane–helium system, and the *Peq* of the THF—99 mol% methane—1 mol% ethane system is close to the *Peq* of the THF–methane system. To quantify the effect of ethane on hydrate formation, the *Peq* for the THF–methane (1)–ethane (2) system is compared with the THF–methane (1) system from the literature [30]. The Δ*P*% between the THF–methane (1)–ethane (2) system (*P_i_*) and THF–methane (1) system (*P_0_*) is depicted in Figure 4. The positive value of the Δ*P*% (Δ*P*% > 0) indicates that ethane has an inhibiting effect on *Peq* under the same temperature and THF concentration. The negative value of the Δ*P*% (Δ*P*% < 0) indicates that under the same temperature and THF concentration, ethane has a promoting effect on *Peq*.

As illustrated in Figure 4, the Δ*P*% between the THF–methane–ethane system (*P_i_*) and the THF–methane system (*P*_0_) is different in different ethane contents and THF concentrations. The trend with respect to temperature is similar to our previous work [30]. The values of Δ*P*% with different temperatures approach a stable value in the same THF solutions. The averages of Δ*P*% are used to represent this stable value, and the standard deviations of Δ*P*% are used to indicate the deviation from the stable value. The standard deviations are caused by the errors between different experimental conditions, which should be less than 2%.

In the high ethane content (*y*_2_ = 10 mol%), the effect of ethane is an inhibiting effect on *Peq*. The averages of Δ*P*% for different THF concentrations from 5 wt% to 19 wt% THF are 12.5%, 9.6% and 11.7%, respectively. The standard deviations of Δ*P*% for different THF concentrations in aqueous solutions from 5 wt% to 19 wt% THF are 1.4%, 1.1%, and 0.8%, respectively. The averages of Δ*P*% are all around 10%, which is the content of ethane in the gas mixture. The differences among the averages of Δ*P*% approach the standard deviations. It can be inferred that the differences among the averages of Δ*P*% can be neglected. It can be inferred that the main effect of 10 mol% ethane on *Peq* is the decrease in the partial pressure (fugacity) of methane in the systems.

In the low ethane content (*y*_2_ = 1 mol%), the Δ*P*% for different THF concentrations from 5 wt% to 19 wt% THF are 0.1%, −1.5%, and 0.4, respectively. The standard deviations of Δ*P*% for different THF concentrations from 5 wt% to 19 wt% THF are 1.8%, 0.5%, and 0.7%, respectively. The differences among the averages of Δ*P*% approach the standard deviations. It can be inferred that the differences among the averages of Δ*P*% can be neglected. It can be inferred that the effect of 1 mol% ethane is weak in the systems.

**Figure 6:**
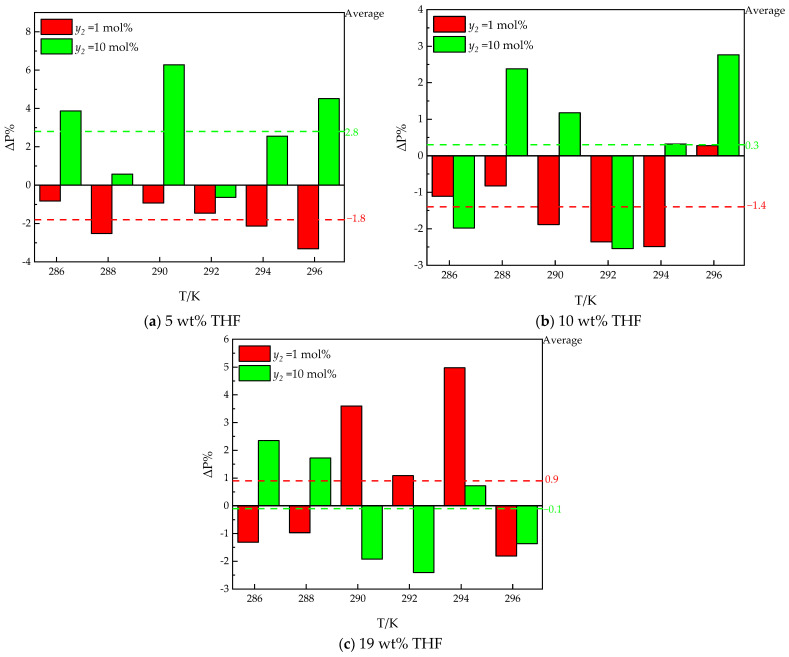
The differences in *Peq* between the THF–methane (1)–ethane (2) system and THF–methane (1)–helium (3) [30].

In conclusion, ethane has weak effects in the THF–methane–ethane system. To further present the effect of ethene, the THF–methane–helium system is used as a comparative system because helium almost does not participate in the formation of hydrate [30]. The Δ*P*% between the THF–methane–ethane system (*P_i_*) and THF–methane–helium system (*P*_0_) is depicted in Figure 6. The positive value of the Δ*P*% (Δ*P*% > 0) indicates that ethane has an inhibiting effect on *Peq*, compared with helium, under the same temperature, methane content, and THF concentration. The negative value of the Δ*P*% (Δ*P*% < 0) indicates that, under the same temperature, methane concentration, and THF concentration, ethane has a promoting effect compared with helium.

As illustrated in Figure 6, the Δ*P*% between the THF–methane–ethane system (*P_i_*) and THF–methane–helium system (*P*_0_) is different in different ethane contents and THF concentrations. The averages and standard deviations of Δ*P*% are also used to evaluate the effects of ethane on *Peq*.

In the low ethane content (*y*_2_ = 1 mol%), the average Δ*P*% is Δ*P*% for different THF concentrations from 5 wt% to 19 wt% THF are −1.8%, −1.4%, and 0.9%, respectively. The standard deviations of Δ*P*% for different THF concentrations from 5 wt% to 19 wt% THF are 0.4%, 0.4%, and 1.0%, respectively. The averages of Δ*P*% are much higher than the standard deviations for 5 wt% and 10 wt% THF systems. It can be inferred that 1 mol% ethane has a promoting effect on hydrate formation compared with helium for 5 wt% and 10 wt% THF systems. But the average of Δ*P*% for the 19 wt% THF system approaches the standard deviation. It can be inferred that the effect of ethane is not statistically significant for 19 wt% THF systems. The promoting effect on *Peq* is decreasing with the increase in THF concentrations.

However, in the high ethane content (*y*_2_ = 10 mol%), the promoting effect of ethane on *Peq* does not work. The average indicators of Δ*P*% for different THF concentrations from 5 wt% to 19 wt% THF are 2.8%, 0.3%, and −0.1%, respectively. The standard deviations of Δ*P*% for different THF concentrations from 5 wt% to 19 wt% THF are 1.0%, 0.8%, and 0.9%, respectively. The average Δ*P*% is much higher than the standard deviations for 5 wt% THF systems. It can be inferred that 10 mol% ethane has an inhibiting effect on hydrate formation compared with helium for 5 wt% THF systems. But the averages of Δ*P*% for 10 wt% and 19 wt% THF systems approach the standard deviation. It can be inferred that the effect of ethane is not statistically significant in the 10 wt% and 19 wt% THF system.

In the THF–methane–ethane system, the averages of Δ*P*% in different THF concentrations and different ethane contents are always less than 3%. Compared with pure methane, the main effect of ethane on *Peq* is the dilution effect on methane. As the mole fraction of ethane in the gas phase increases, ethane dilutes the mole fraction of methane in the gas phase and reduces the fugacity of CH_4_, thus increasing the *P_eq_* of hydrate formation. However, compared with the THF–methane–helium system, the dilution effect of ethane on methane is not the whole effect on *Peq*. The effect of ethane on *Peq* is the same as in the literature [34]. Raman spectroscopic data in the literature [34] reported that THF hydrate and ethane hydrate have a competitive effect in high THF concentrations, with less ethane hydrate being formed in systems with higher THF concentrations. The competitive effect on *Peq* in low THF concentrations (5 wt%) of this work is the same as high THF concentrations in the literature [34]. *Peq* is inhibited by high ethane content (*y*_2_ =10 mol%) in low THF concentrations (5 wt%). However, the effect of 1 mol% ethane on *Peq* decreases, promoting the effect in the systems with the increase in THF concentrations compared with helium.

### 3.2. The Effects of Ethane on Hydrate Formation in the THF–Methane–Ethane–Helium–Carbon Dioxide System

**Figure 7:**
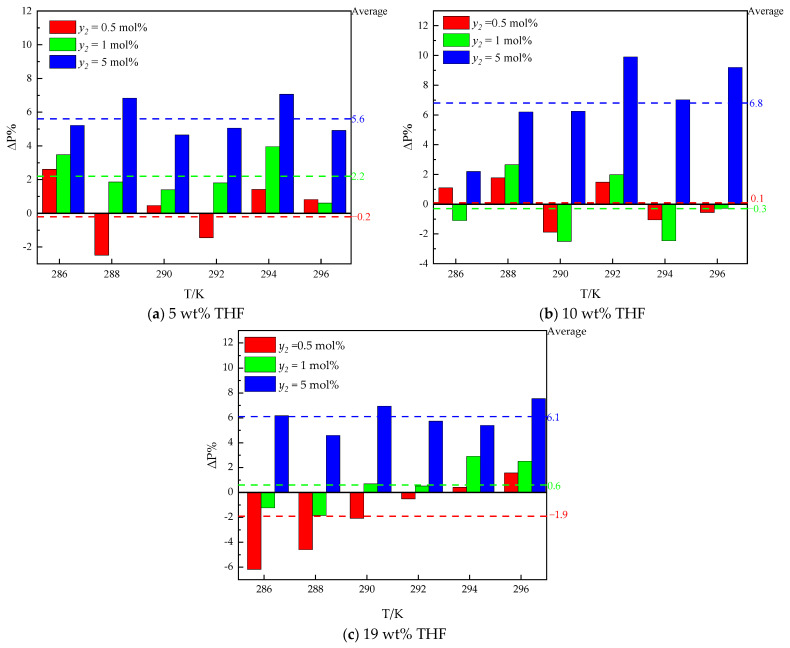
The differences in *Peq* between systems with and without ethane in the presence of THF.

To study the effects of ethane on hydrate formation, the *Peq* in the THF–methane–helium–carbon dioxide system from the literature [30] is introduced for comparison. The Δ*P*% in the presence (as *P_i_*) and absence (as *P*_0_) of ethane shows the effects of ethane on *Peq*, which is depicted in Figure 7. The positive value of the Δ*P*% (Δ*P*% > 0) indicates that under the same temperature, helium content, carbon dioxide content, and THF concentration, ethane has an inhibiting effect on *Peq* compared with a system in the absence of ethane. The negative value of the Δ*P*% (Δ*P*% < 0) indicates that under the same temperature, helium content, carbon dioxide content, and THF concentration, ethane has a promoting effect compared with a system in the absence of ethane.

As illustrated in Figure 7, the difference in *Peq* between the systems in the absence of ethane (*y*_2_ = 0) and the presence of ethane (*y*_2_ = 0.5–5 mol%) is different among different THF concentrations. The averages and standard deviations of Δ*P*% are also used to evaluate the effects of ethane on *Peq*.

In the 5 wt% THF aqueous solution system, the averages of Δ*P*% for systems in the presence of 0.5 mol%, 1 mol%, and 5 mol% ethane are −0.1%,2.2%, and 5.6%, respectively. And the standard deviations of Δ*P*% are 0.9%, 0.5%, and 0.5%, respectively. The average of Δ*P*% for 0.5 mol% ethane systems approaches the standard deviation. It can be inferred that the effect of ethane is not statistically significant for 0.5 mol% ethane systems. However, the averages of Δ*P*% for 1 mol% and 5 mol% ethane systems are much higher than the standard deviations. It can be found that the effect of ethane on hydrate formation increases *Peq*. It can be inferred that 1 mol% and 5 mol% ethane contents have an inhibiting effect on hydrate formation compared with systems in the absence of ethane (*y*_2_ = 0).

In the 10 wt% THF aqueous solution system, the averages of Δ*P*% for systems in the presence of 0.5 mol%, 1 mol%, and 5 mol% ethane are 0.1%, −0.3%, and 6.8%, respectively. And the standard deviations of Δ*P*% are 0.6%, 0.8%, and 1.1%, respectively. The averages of Δ*P*% for 0.5 mol% and 1 mol% ethane systems approach the standard deviations. It can be inferred that the effect of ethane is not statistically significant for 0.5 mol% and 1 mol% ethane systems. However, the averages of Δ*P*% for the 5 mol% ethane THF system are much higher than the standard deviations. In 19 wt% THF aqueous solution, the averages of Δ*P*% for systems in the presence of 0.5 mol%, 1 mol%, and 5 mol% ethane are −1.9%, 0.6%, and 6.1%, respectively. And the standard deviations of Δ*P*% are 1.4%, 0.9%, and 0.6%, respectively. The averages of Δ*P*% for 0.5 mol% and 1 mol% ethane systems approach the standard deviations. It can be inferred that the effect of ethane is not statistically significant for 0.5 mol% and 1 mol% ethane systems. However, the averages of Δ*P*% for the 5 mol% ethane system are much higher than the standard deviations. It can be inferred that 5 mol% ethane has an inhibiting effect on hydrate formation compared with systems in the absence of ethane (*y*_2_ = 0). It can be inferred that 5 mol% ethane has an inhibiting effect on all THF aqueous solution systems on hydrate formation compared with systems in the absence of ethane (*y*_2_ = 0). 0.5 mol% ethane systems have no significant effect on *Peq*.

The effect of ethane on *Peq* for the THF–methane–ethane–helium–carbon dioxide system is similar to the THF–methane–ethane system. The competitive effect on *Peq* for 5 mol% ethane in the THF–methane–ethane–helium—carbon dioxide system is the same as 1 mol% ethane in the THF–methane–ethane system. The promoting effect on *Peq* for 0.5 mol % ethane and 19 wt% THF concentration in the THF–methane–ethane–helium–carbon dioxide system is the same as the 1 mol% ethane in the THF–methane–ethane system. The differences between the two systems in ethane contents and THF concentrations are that the methane content is lower, and carbon dioxide is introduced into the system. It can be inferred that ethane and THF have a competitive effect on hydrate formation. The high ethane content (5 mol%) can increase the *Peq*, and the low ethane content (<1 mol%) has no significant effect on *Peq* (<3% of *Peq* in the absence of ethane).

### 3.3. The Effects of Ethane on Hydrate Formation in This Model

**Figure 8:**
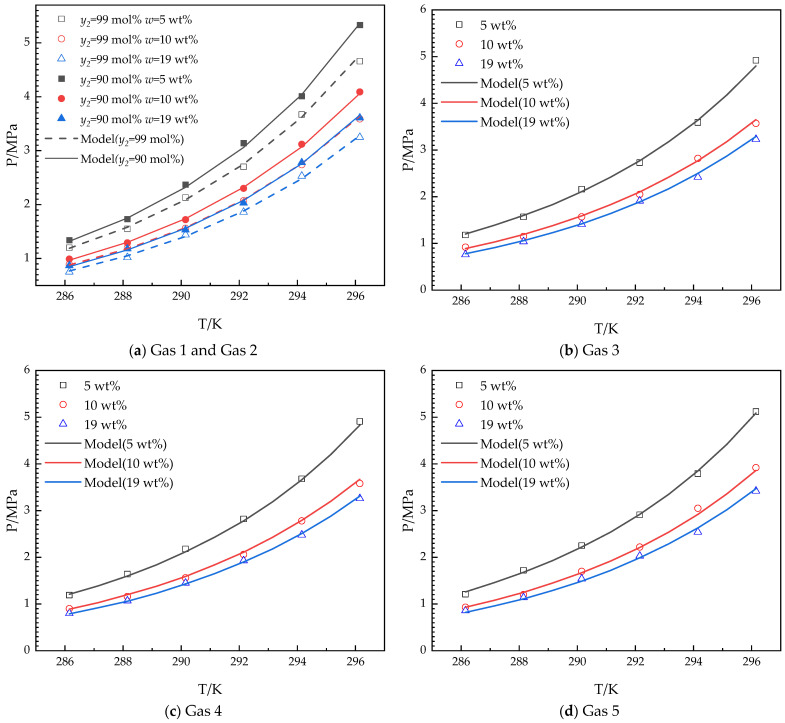
Experimental and model-predicted equilibrium hydrate formation conditions for different systems.

The novel thermodynamic model for predicting *Peq* in this work is based on the Chen–Guo model [39]. The experimental and model-predicted results in the THF–methane–ethane system and THF–methane–ethane–helium–carbon dioxide system are shown in Figure 8.

As shown in Figure 8, the proposed thermodynamic model agrees well with the experimental *Peq* values for three different THF concentrations (5.0, 10.0, and 19.0 wt%) in the aqueous solutions and different gases (Gas 1–Gas 5). The accuracy of the model in this work is evaluated by average relative deviation (*ARD*) and goodness of fit (*GF*). ARD and GF are used to calculate the deviation between experimental (exp) and predicted (pre) *Peq*.

(4) $$ARD=\sum_{\mathrm{ii}\;}^{n}\left|\frac{P_{\mathrm{eq},\;\exp,\mathrm{ii}\;}-P_{\mathrm{eq},\;\mathrm{pre},\mathrm{ii}\;}}{P_{\mathrm{eq},\;\exp,\mathrm{ii}\;}}\right|/n\cdot 100\%$$

(5) $$GF=1-\frac{\sum_{ii}^{n}{\left(P_{eq,exp,ii}-P_{eq,pre,ii)}\right)}^{2}}{\sum_{ii}^{n}{\left(P_{eq,\;\exp,\mathrm{ii}\;}-\sum_{ii}^{n}P_{eq,pre,ii}/n\right)}^{2}}$$

where $P_{\mathrm{eq},\exp}$ is the experimental thermodynamic equilibrium hydrate formation pressure. $P_{\mathrm{eq},p\mathrm{re}}$ is the predicted thermodynamic equilibrium hydrate formation pressure. *n* is the number of experiments. *ii* is the experiment serial number. The *ARD*s and *GF* between experimental and predicted *Peq* are listed in Table 2.

As shown in Table 2, all the *ARD*s for all the systems are less than 3%, and GF is more than 0.998. It confirms that the accuracy of the model in this work meets the prediction of *Peq*. According to the competitive effect of ethane and THF on *Peq* (described in Section 3.1 and Section 3.2), the Chen–Guo model [39] has been modified as the proposed model to adapt to the effects. The proposed model accurately describes the correlation well.

The proposed model extends the THF concentration range of the Chen–Guo model [34] for the competitive effect of ethane on *Peq* (details in Section 4.3). The effects of low THF concentrations (5–19 wt%) on *Peq* can be better quantified using the proposed model. The phase equilibrium conditions of the systems in this work are determined by the difference in chemical potential between phases (Δ*μ*). The Δ*µ* expresses how the competitive effect varies with THF concentration and ethane content, helping further understand the competitive effect of ethane on *Peq* and can guide the selection of the system with the THF concentrations and ethane content.

## 4. Materials and Methods

### 4.1. Materials

The HNGs were provided by Beijing Yongsheng Gas Industry Company (Beijing, China). The HNG systems (mixture of methane, ethane, carbon dioxide, and helium) were measured in this work, as listed in Table 1. All the mole ratios of the gases are the same as in the cylinder. The uncertainty of the mole ratio is ±0.05 mol%. THF (purity ≥ 99%) was provided by Shanghai Denou Chemical Company (Shanghai, China). The deionized water (18 × 10^6^ Ω·cm) and THF were weighed by an electronic balance (±0.1 mg). The initial THF concentrations were 5, 10, and 19 wt%. In 19 wt% THF aqueous solution, the mole ratio of THF to water is 1/17, which is the same as the mole ratio of THF to the water of sII hydrate (1/17) [40]. The uncertainty of the THF concentrations is ±0.1 wt%.

**Figure 9:**
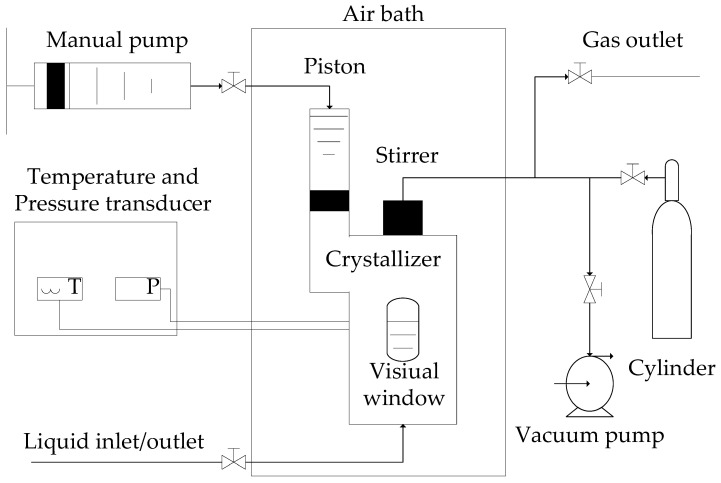
Schematic diagram of experimental apparatus for the measurements of the equilibrium hydrate formation conditions.

The experimental apparatus in this work is the same as in our previous work [30], as shown in Figure 9. The temperature range of the crystallizer is 253.15 to 323.15 K and is controlled by an air bath. The maximum pressure of the crystallizer is 20.00 MPa. The crystallizer was regulated by a hand pump with a maximum volume of 465.0 mL. The uncertainties of the measured pressure and temperature were ±0.005 MPa and ±0.05 K, respectively.

### 4.2. Experimental Methods

**Figure 10:**
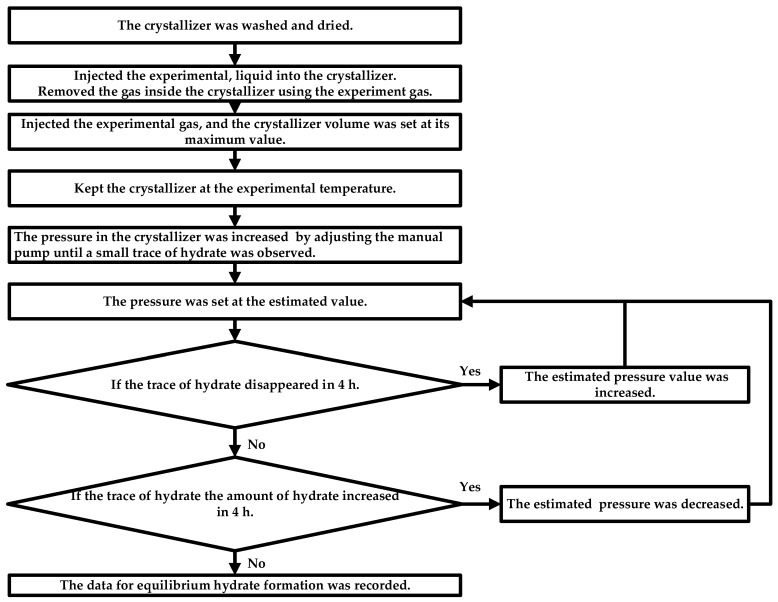
The experimental procedure for investigating the *Peq* through the isothermal pressure search method [30].

The pressure search method was used to measure *Peq* of different initial THF concentration systems in this work [30]. The experimental setup for this study is shown in Figure 10 and is the same as that used in the previous study [30,41].

### 4.3. Model Methods

The novel thermodynamic model for predicting *Peq* in this work is based on the Chen–Guo model [39]. Patel–Teja (PT) equation of state (EoS) is used to calculate the fugacity of the gas phase [42]. PT EoS is an accurate method for gas–hydrate phase equilibria for ethane [41]. The Wilson activity coefficient model is used to calculate the fugacity of the liquid phase. The Wilson activity coefficient model is a simple and accurate method for gas–liquid phase equilibria of nonionic solutions [30]. The calculation of PT EoS and Wilson activity is provided in the Supplementary Materials.

The Chen–Guo model is a two-step hydrate formation. The first step is a quasi-chemical reaction process to form basic cages of hydrate. The second step is an adsorption process to form linked cages of hydrate. THF and ethane are trapped in basic cages. Methane, carbon dioxide, and helium are trapped in linked cages. In this work, the binary interaction parameter between gases in linked cages and basic cages and the chemical potential criterion (Δ*μ*) of double hydrate (THF hydrate and ethane hydrate) are introduced in the new model. In the new model, the effect of ethane in HNGs on *Peq* is accounted for and can be quantitatively described in this thermodynamic framework. It predicts the *Peq* of the HNGs in this work accurately.

**Figure 11:**
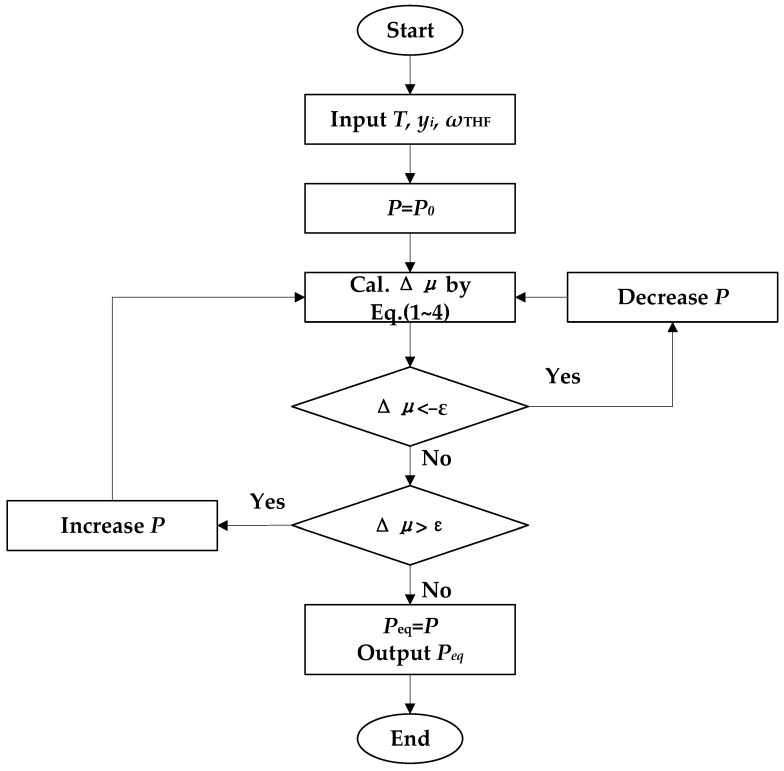
Schematic diagram of the prediction of *Peq.* $y_{i}$ is the mole fraction of gases in the gas phase; *w* is the mass fraction of THF, and ε is the maximum deviation in Δμ (10^−5^ in this work) [41].

The phase equilibrium conditions of the systems in this work are determined by the difference in chemical potential between phases, which are liquid–hydrate phase for $\Delta \mu_{THF}$ and gas–hydrate phases for ethane ($\Delta \mu_{C_{2}H_{6}}$). When the Δμ = 0, the pressure of the system is the *Peq* [41,43]. The procedure for predicting *Peq* is illustrated in Figure 11.

Based on the Chen–Guo model [39], the Δ*µ* of hydrate mixtures can be described as follows:

(6) $$\Delta \mu =X_{C_{2}H_{6}}\;\Delta \mu_{C_{2}H_{6}}+X_{THF}\Delta \mu_{THF}$$

(7) $$X_{C_{2}H_{6}}+X_{THF}=1$$

(8) $$\Delta \mu_{THF}=\mathrm{RT}\left[\lambda_{2}\ln\frac{f_{THF}^{H}}{f_{THF}}+\sum_{i}\lambda_{1}\ln\left(1-\theta_{i}\right)\right]$$

(9) $$\Delta \mu_{C_{2}H_{6}}=\mathrm{RT}\left[\lambda_{2}\ln\frac{f_{C_{2}H_{6}}^{H}}{f_{C_{2}H_{6}}}+\sum_{i}\lambda_{1}\ln\left(1-\theta_{i}\right)\right]$$

where $X_{C_{2}H_{6}}$ and $X_{THF}$ is the mole fraction of ethane and THF basic hydrate, respectively. $\theta_{i}$ is the occupation fraction of the linked cages in hydrates filled with gas, and *i* is the CH_4_, CO_2_, and helium, respectively. $f_{THF}$ and $f_{THF}^{H}$ are the fugacity of THF in the liquid phase and the basic hydrate under the experimental condition, respectively. $f_{C_{2}H_{6}}$ and $f_{C_{2}H_{6}}^{H}$ are the fugacity of ethane in the liquid phase and basic hydrate under the experimental condition, respectively. R is the gas constant (8.314 J·K^−1^·mol^−1^). $\lambda_{1}$ is the ratio of the linked-cage number to the water-molecule number. $\lambda_{2}$ is the ratio of the basic-cage number to the water-molecule number. $\lambda_{1}$ and $\lambda_{2}$ are determined by the hydrate structure (sII). For each of THF or ethane hydrate (sII), $\lambda_{1}$ is 2/17 and $\lambda_{2}$ is 1/17 [40].

$\theta_{i}$ is used to describe the second step of Langmuir adsorption, and it can be expressed as follows [41]:

(10) $$\theta_{i}=\frac{f_{i}C_{i}}{1+\sum_{i}\left(f_{i}C_{i}\right)}$$

where $f_{i}$ is the fugacity of gases calculated by PT EOS. $C_{i}$ is the Langmuir constant of CH_4_, CO_2_, and helium correlated as an Antoine-type equation [29]:

(11) $$C_{i}=X^{\prime }\exp\left(\frac{Y^{\prime }}{T-Z^{\prime }}\right)$$

where $X^{\prime }$, $Y^{\prime }$, and $Z^{\prime }$ are the Antoine parameters. The parameters for CH_4_, CO_2_, and helium are fitted by the experimental data of this work. The parameters are fitted based on the *Peq* from the literature and, in this study, by trial-and-error method [43], as shown in Table 3.

The fugacity of THF and ethane in the hydrate phases is used to describe the first step of the process and is calculated as follows [39]:

(12) $$f_{THF}^{H}=f_{T}^{H}\left(T\right)\cdot \exp\left(\frac{\beta P}{T}\right){\cdot \alpha }_{w}^{-\frac{1}{\lambda_{2}}}$$

where *β* is the parameter of hydrate structure, which is 10.244 K/MPa [40]. $\alpha_{w}$ is the activity of the water calculated by the Wilson model. $f_{T,j}^{H}\left(T\right)$ is a faction of temperature can be written as follows [29]:

(13) $$f_{T}^{H}(T)=exp(\frac{{\sum_{i}A}_{i}\theta_{i}}{T})\cdot A^{\prime }exp(\frac{B^{\prime }}{T-C^{\prime }})$$

where $A^{\prime }$, $B^{\prime }$, and $C^{\prime }$ are the Antoine parameters. The $A_{i}$ is the binary interaction parameter between the gas (CH_4_, CO_2_, and helium) and THF or $C_{2}H_{6}$ in the hydrate. The parameters are fitted based on the *Peq* from the literature and, in this study, by the trial-and-error method [43]. The parameters are listed in Table 4.

## 5. Conclusions

This work explored the equilibrium hydrate formation conditions of THF–methane–ethane and THF–methane–ethane–helium–carbon dioxide systems. The effects of ethane on *Peq* were quantitatively described in this work. A new thermodynamic model was proposed to predict the *Peq* of double hydrate (ethane hydrate and THF hydrate) and applied in the two systems. This model can accurately predict the *Peq* in ethane–THF hydrate systems. The *ARD*s are less than 3%, and *GF*s for HNGs–THF–water systems are more than 0.998. In the THF–methane–ethane systems, the main effect of ethane on *Peq* is the dilution effect on methane. As the ethane content in the gas phase increases, the methane content decreases and reduces the fugacity of methane, thus increasing the *P_eq_*. In addition to the dilution effect on methane, ethane, and THF have a competitive effect on hydrate formation. 10 mol% ethane content in the gas phase can decrease in *Peq* in different THF concentration solutions. In the THF–methane–ethane–helium–carbon dioxide systems, the same dilution effect on methane still exists in the THF–methane–ethane systems. The 5 mol% ethane content in the gas phase can increase the *Peq* by more than 5%. Ethane and THF have the same competitive effect on hydrate formation as the THF–methane–ethane system. The low ethane content (<1 mol%) has no significant effect on *Peq* (<3% of *Peq* in the absence of ethane). The effects can help to further understand the effects of ethane on *Peq* and can guide the prediction and control of HBGS extracting helium from HNGs. This work showed the feasibility of HBGS, and the separation effect needs to be further studied in our next work.

## Figures and Tables

**Table 1:** Composition of individual mixtures.

Gas	$CH_{4}$ (mol%)	$C_{2}H_{6}$ (mol%)	He (mol%)	${CO}_{2}$ (mol%)
	*y*_1_	*y*_2_	*y*_3_	*y*_4_
Gas 1	99.0	01.0	-	-
Gas 2	90.0	10.0	-	-
Gas 3	95.1	00.5	0.4	4.0
Gas 4	94.6	01.0	0.4	4.0
Gas 5	90.6	05.0	0.4	4.0

The uncertainty of the mole ratio is ±0.05 mol%.

**Table 2:** The *ARD*s and *GF*s for all the systems in this work.

Gases	*ARD*	*GF*
Gas 1	1.6%	0.999
Gas 2	1.5%	0.999
Gas 3	2.1%	0.999
Gas 4	1.7%	0.999
Gas 5	2.2%	0.999

**Table 3:** The parameters of $C_{i}$ used for this model.

$C_{i}$	*X*′ (Pa)	*Y*′ (K)	*Z*′ (K)
$C_{1}\;({CH}_{4})$	6.2728 × 10^−15^	4879.29	23.01
$C_{2}\;({CO}_{2})$	1.6464 × 10^−11^	2799.66	15.90
$C_{3}\;(helium)$	6.0000 × 10^−12^	2034.89	6.31

**Table 4:** The parameters of $f_{T}^{H}$ used for this model.

$f_{T}^{H}$	$A^{\prime }$ (Pa)	$B^{\prime }$ (K)	$C^{\prime }$ (K)	$A_{{CH}_{4}}$	$A_{{CO}_{2}}$	$A_{He}$
THF	1.8 × 10^24^	−2.00 × 10^4^	−130	300	300	100
$C_{2}H_{6}$	7.7 × 10^16^	−5.56 × 10^3^	− 57.9	300	300	150

## Data Availability

The original contributions presented in this study are included in the article/Supplementary Materials. Further inquiries can be directed to the corresponding author.

## Supplementary Materials

The following supporting information can be downloaded at: https://www.mdpi.com/article/10.3390/molecules30102109/s1 [44], Figure S1:The pressure-temperature flash procedure for calculating $P_{2}^{sat}$; Table S1: Parameters of PT EOS.
