# Supplementary material for: The Experimental and Modeling Study on the Effect of Ethane in Helium-Rich Natural Gas on the Thermodynamic Equilibrium of Hydrate Formation in the Presence of Tetrahydrofuran
Source: Molecules. 2025 May 9;30(10):2109. doi: 10.3390/molecules30102109 (PMC12114356; doi:10.3390/molecules30102109)
Supplement: Supplementary file 1 [file molecules-30-02109-s001.zip › molecules-3579298-supplementary.pdf]

Supplementary Materials S1 Calculation for the Fugacity

The PT EoS to calculate the fugacity of gases, which expressed as follows[1] :

$$P = \frac{RT}{v-b} - \frac{a[T]}{v(v+b)+c(v-b)} \quad \text{(S1)}$$

$$a(T) = \frac{\Omega_a \alpha(T) R^2 T_c^2}{P_c} \quad \text{(S2)}$$

$$b = \frac{\Omega_b R T_c}{P_c} \quad \text{(S3)}$$

$$c = \frac{\Omega_c R T_c}{P_c} \quad \text{(S4)}$$

$$\Omega_a = 3{-}3\zeta^2 + 3(1-2\zeta)\Omega_b + \Omega_b^2 + 1 - 3\zeta \quad \text{(S5)}$$

$$\Omega_b^3 + (2-3\zeta)\Omega_b^2 + 3{-}3\zeta^2\Omega_b - \zeta^3 = 0 \quad \text{(S6)}$$

$$\Omega_a = 1 - 3\zeta \quad \text{(S7)}$$

$$\alpha = [1 + F(1 - T_r^{0.5})]^2 \quad \text{(S8)}$$

where ζ and F are the constants associated with the gas molecule in the PT EoS, and the values are listed in Table S1.

Table S1. Parameters of PT EOS[1]

Gases	ζ	F
CH_4	0.324	0.455336
CO_2	0.309	0.707727
helium	0.329	0.452413
THF	0.313	0.733317

The P_2^{sat} is calculated by pressure–temperature flash using PT EoS. The procedure is shown in Figure S1.

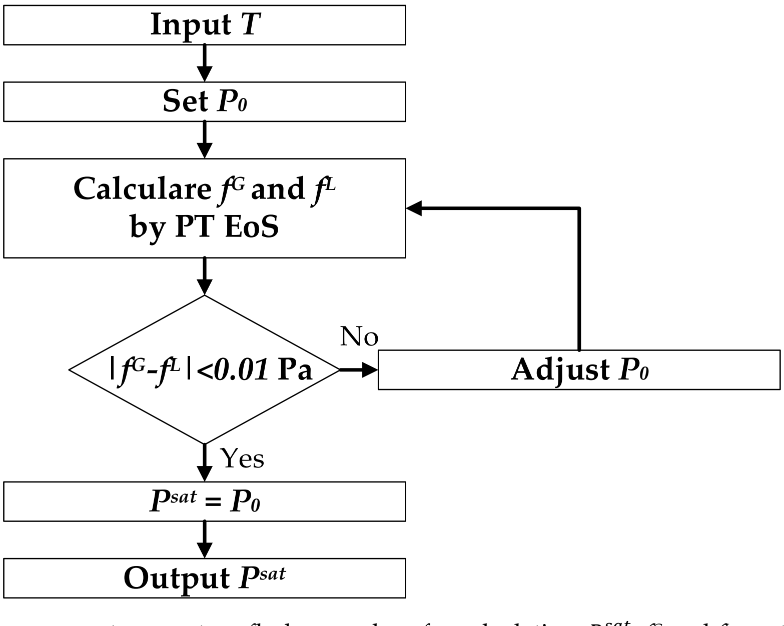

Figure S1. The pressure-temperature flash procedure for calculating P_2^{sat}. f^G and f^L are the fugacity of THF in gas and liquid phase in a pure THF system[2].

The activity of water in aqueous solution was calculated by the Wilson model to be written as follows[3] :

$$\ln\,\gamma_1 = -\ln\,(x_1 + \Lambda_{12}x_2) + x_2\left(\frac{\Lambda_{12}}{x_1 + \Lambda_{12}x_2} - \frac{\Lambda_{21}}{x_2 + \Lambda_{21}x_1}\right) \quad \text{(S9)}$$

$$\ln\,\gamma_2 = -\ln\,(x_2 + \Lambda_{21}x_1) + x_1\left(\frac{\Lambda_{21}}{x_2 + \Lambda_{21}x_1} - \frac{\Lambda_{12}}{x_1 + \Lambda_{12}x_2}\right) \quad \text{(S10)}$$

$$\Lambda_{12} = \frac{v_2^{\mathrm{L}}}{v_1^{\mathrm{L}}}\exp\left(-\frac{\lambda_{12} - \lambda_{11}}{RT}\right) \quad \text{(S11)}$$

$$\Lambda_{21} = \frac{v_1^{\mathrm{L}}}{v_2^{\mathrm{L}}}\exp\left(-\frac{\lambda_{21} - \lambda_{22}}{RT}\right) \quad \text{(S12)}$$

$$\alpha_w = \gamma_1 x_1 \quad \text{(S13)}$$

where x_1 and x_2 are the mole fractions of water and THF, respectively. v_2^{L} and v_1^{L} are the mole volumes of water and THF, respectively. The mole volumes of water and THF can be calculated by fitting polynomials from the literature [4]. γ_1 and γ_2 are the activity coefficients of water and THF, respectively. $\lambda_{12} - \lambda_{11}$ is 1865.2097 J/mol and $\lambda_{21} - \lambda_{22}$ is 1927.6307 J/mol according to the literature [3].

The fugacity of THF in liquid is corrected by polynomials as follows[2]:

$$f_{THF} = \gamma_2 x_2 P_2^{sat} exp\,(\frac{v_2^{\mathrm{L}}(p - P_2^{sat})}{RT}) f_{cor} \quad \text{(S14)}$$

$$f_{cor} = -8.7774w^2 + 0.444w + 1.3176 \quad \text{(S15)}$$

where P_2^{sat} is the saturated vapor pressure of THF. w is the mass fraction of THF in the aqueous solution. f_{cor} is fitted by *Peq* from the literature[2] and this study.

References

1. Patel, N.C.; Teja, A.S. A new cubic equation of state for fluids and fluid mixtures. *Chem. Eng. Sci.* **1982**, 463-473. https://doi.org/10.1016/0009-2509(82)80099-7.
2. Liu, Z.; Zhang, G.; Lu, F.; Ren, Q.; Xu, Z.; Fan, S.; Sun, Q.; Wang, Y.; Guo, X. The Experimental and Modeling Study on the Thermodynamic Equilibrium Hydrate Formation Pressure of Helium-Rich Natural Gas in the Presence of Tetrahydrofuran. *Molecules* **2024**, *29*, 4827. https://doi.org/10.3390/molecules29204827.
3. Sun, Q.; Guo, X.; Chapman, W.G.; Liu, A.; Yang, L.; Zhang, J. Vapor – hydrate two-phase and vapor – liquid – hydrate three-phase equilibrium calculation of THF/CH4/N2 hydrates. *Fluid Phase Equilib.* **2015**, *401*, 70-76. https://doi.org/10.1016/j.fluid.2015.05.024.
4. Ivanov, E.V. To the issue of temperature-dependent behavior of standard molar volumes of components in the binary system (water+tetrahydrofuran) at ambient pressure. *The Journal of Chemical Thermodynamics* **2014**, *72*, 37-43. https://doi.org/10.1016/j.jct.2013.12.028.
